# Addressing Oral Health Inequities, Access to Care, Knowledge, and Behaviors

**DOI:** 10.5888/pcd18.210060

**Published:** 2021-03-25

**Authors:** Casey J. Hannan, Timothy L. Ricks, Lorena Espinoza, Jane A. Weintraub

**Affiliations:** 1 National Center for Chronic Disease Prevention and Health Promotion, Centers for Disease Control and Prevention, Atlanta, Georgia; 2 US Public Health Service, Indian Health Service Headquarters, Division of Oral Health, Rockville, Maryland; 3 University of North Carolina at Chapel Hill, Adams School of Dentistry and Gillings School of Global Public Health, Chapel Hill, North Carolina

Oral health is essential to overall health (1), and dental public health is a field of public health and a specialized field of dentistry that focuses on improving access to oral health care and understanding the factors that contribute to improving oral health from a population health perspective (2). This collection of articles in *Preventing Chronic Disease* (PCD), “Oral Health Behaviors and Availability of Dental Services Among Children and Adults,” features 8 articles that discuss contemporary dental public health challenges and opportunities. These include inequities in access to dental care, disparities in the prevalence of oral disease, risk behaviors related to oral disease, the relationship between oral health and chronic diseases, and the effect of the COVID-19 pandemic on oral health.

Healthy People 2030 is the fifth iteration of national health objectives for the United States, and like previous editions, includes oral health (3). These objectives are categorized by health conditions, health behaviors, and populations, and the 11 that deal with oral health serve as a framework for articulating how findings and recommendations presented in this PCD collection align with established strategies to improve national oral health outcomes.

Healthy People 2030 oral health objective 6 is to reduce the proportion of adults aged 45 years or older with moderate or severe periodontitis, a disease linked to chronic diseases such as diabetes (4), adverse pregnancy outcomes (5), atherosclerotic cardiovascular disease (6), rheumatoid arthritis (7), Alzheimer’s disease (8), chronic obstructive pulmonary diseases (9), nonalcoholic fatty liver disease (10), and others. In the article by Seitz et al, “Current Knowledge on Correlations Between Highly Prevalent Dental Conditions and Chronic Diseases: An Umbrella Review,” the authors examined 1,249 systematic reviews on the relationships between oral diseases and chronic diseases and included 32 in their qualitative synthesis (11). They found that periodontitis was the dental condition most frequently correlated with chronic systemic diseases. Conversely, type 2 diabetes was the chronic systemic disease that had the most frequently observed correlations with dental conditions. Most dental–chronic disease correlations were found between periodontitis and diabetes and between periodontitis and cardiovascular disease. The authors suggest that these correlations should be factored into care plans for people with comorbid or multimorbid dental and chronic disease conditions. They also suggest that more awareness is needed about evidence on correlations between dental conditions and chronic diseases and potential opportunities for medical–dental integration in delivery of care. They highlight the need for more research on the causal links between dental conditions and chronic diseases. In addition, longitudinal research studies are needed to document the direction of causality between oral health and systemic diseases.

Access to dental care for prevention and treatment is critical to ensure optimal oral health. Healthy People 2030 oral health objective 8 is to increase the proportion of children, adolescents, and adults who use the oral health care system. Increasing use of the oral health system is also a Leading Health Indicator, a small subset of high-priority Healthy People 2030 objectives selected to drive action toward improving health and well-being in the United States (12). Unfortunately, access to oral health care is a challenge: only 43% of the US population had a dental visit in 2015 (13). Moreover, some segments of the population — certain racial/ethnic minority groups, people living in poverty, and people living in rural areas — have even less access to dental care. Overall, people of all ages living in rural America have about 8% (children aged ≥2 y) to 10% (adults aged 18–64 y) less access to dental services compared with their urban counterparts. Children in rural areas are 5% less likely to receive preventive dental care than children in urban areas, and adults in rural areas are 7% more likely to have missing teeth (14).

Two articles in this collection address Healthy People 2030’s oral health objective 8 by using data from predominantly rural states, Alabama and Georgia. In “Visualizing County-Level Data to Target Dental Safety-Net Programs for Children,” Hamilton et al used geographic information systems (GIS) to showcase visually how dental safety-net clinics in Georgia were placed in the areas of highest need (15). Their methodology can be used by others to help assess whether dental public health resources are allocated in places of greatest need, that is, where the prevalence of untreated caries is high. The data used to generate the maps are publicly available for all states and, thus, could enable any jurisdiction to replicate the assessment.

In “Visualizing Potential Effects of Dentist Retirements on Accessibility to Dental Care Among Children in Alabama,” Samsel et al also used GIS to visualize the effect of dentist retirements on access to oral health care, a topic not previously studied (16). These authors measured the spatial accessibility of a licensed dentist among young people (children, adolescents, and young adults aged ≤20). Rates of access to dentists in this population were higher in urban areas (1.3 providers per 1,000 young people) than in rural areas (0.8 providers per 1,000 young people). The effect of dentist retirements on accessibility to dental care in rural areas was greater than in urban areas. Although the results pertain to Alabama, any state can use these methods to study the effect of dentist retirements on accessibility to care. Strategies to make it easier to get dental care are critical for better oral health and overall health outcomes.

Healthy People 2030 oral health objectives 1 and 2 aim to reduce the proportion of children and adolescents with lifetime tooth decay and children and adolescents with active and untreated tooth decay, respectively. Disparities in access to dental services directly relate to disparities in the prevalence of children and adolescents with these problems. A Centers for Disease Control and Prevention report in 2019 highlighted many of these disparities, showing, for example, that the prevalence of caries and untreated tooth decay among African American and Mexican American children, adolescents, and young adults aged 2 to 19 years was up to 2 to 3 times higher than among their non-Hispanic White counterparts. That report also showed that children and adolescents living below 200% of the federal poverty level had almost double the prevalence of caries and untreated decay as children and adolescents living at or above 200% of the federal poverty level (17).

Healthy People 2030 objective 8 is also relevant in “Racial/Ethnic Disparities Among US Children and Adolescents in Use of Dental Care” (18). Robison et al examined changes in racial/ethnic disparities in annual dental care use among children and adolescents aged 2 to 17 years from 2001 to 2016. With a sample of 132,763 children and adolescents, the researchers found that the gap between dental care use among Hispanic or Latino, Asian, and Black/African American children and dental care use among non-Hispanic White children had narrowed significantly from 2001 to 2016. The disparity in the prevalence of dental care use between non-Hispanic White children and adolescents and Asian children and adolescents declined 75%, from an 18.8 percentage-point difference in 2001 to a 4.7 percentage-point difference in 2016. Among Hispanic/Latino children and adolescents, this disparity declined by 61% (from a 23.6 percentage-point difference to a 9.1 percentage-point difference), and among Black/African American children and adolescents, it declined by 38% (from a 25.4 percentage-point difference to a 15.7 percentage-point difference). By income level, children and adolescents from low-income households of all races/ethnicities showed the most marked increase in use of dental care, increasing by 18% from the 2001–2005 data cycle to the 2011–2016 data cycle. Furthermore, use of dental services among Hispanic/Latino and Asian children and adolescents from low-income households was similar to use among non-Hispanic White children and adolescents but was well below that of children and adolescents from middle- and high-income households, and disparities persisted for Black/African American children and adolescents at all income levels.

Healthy People 2030 oral health objective 9 is to increase the proportion of young people from low-income households who have a preventive care dental visit. In “Oral Health Behaviors in Very Young Children in Low-Income Urban Areas in Chicago, Illinois, 2018–2019,” Martin et al analyzed the oral health behaviors of children from low-income households who were aged 3 years or younger, an age group not studied often for oral health behaviors (19). Important parental and caregiver behaviors included bringing young children to their dental visits and supervising children’s oral hygiene at home. Using caregiver-reported data from 420 families in Cook County, Illinois, and objectively measured plaque index scores, researchers identified correlations between infant and toddler risk factors for oral disease and the oral health of their caregivers. Caregivers who brush their teeth were more likely to brush their children’s teeth as well, and having additional caregivers assist with brushing the child’s teeth was associated with both higher brushing frequency and lower plaque scores. This study points to the need to evaluate the family support structure when assessing risk factors for oral disease in young children, and for oral health professionals to promote additional caregiver and family support in implementing an oral health regimen among infants and toddlers. Creative interventions that facilitate behavior change in parents may help lower the risk for development of dental caries in children.

Another article, “Does Preventive Care Reduce Severe Pediatric Dental Caries?” also examined preventive dental care in children from low-income households (20). Lee et al compared the effect of increased Medicaid reimbursements for preventive dental care on use of tertiary oral health services (caries-related surgery, sedation, and emergency department visits) in children aged 9 years or younger in Texas and Florida. The observational study used Medicaid enrollment and claims filed in 2007 and 2011–2012. Linear regression models estimated the outcomes of preventive care dental visits, caries-related sedations, caries-related emergency department visits, and caries-related surgeries. Examining records of 7,748,850 children, the authors found that after Medicaid reform to increase reimbursement for dental care providers in Texas, preventive care dental visits increased by 11.4%, caries-related surgeries increased by 0.01%, caries-related sedation increased by 1.7%, and caries-related emergency department visits decreased by 0.3%. The authors concluded that increasing provider reimbursements was effective in increasing access to preventive dental care for Medicaid-enrolled children, and although increased prevention resulted in increased procedures to treat caries, the decline in caries-related emergency department visits attributed to prevention quantified the gap in previously unmet need.

Preventive dental care can significantly improve oral health in children. One preventive strategy is the placement of dental sealants, and Healthy People 2030 oral health objective 10 is to increase the proportion of children and adolescents who receive dental sealants on 1 or more of their primary or permanent molars. The chewing surfaces of the molars, known as the occlusal surfaces, are the most susceptible to decay. Among children aged 6 to 11 years with at least 1 decayed tooth, 90% of the disease is located in the first molars; among children and adolescents aged 12 to 17, 79% of disease is located the first and second molars (21). The deep pits and fissures on these surfaces are difficult to clean; dental sealants are directly applied to these surfaces to protect them (22).

In “Awareness Among US Adults of Dental Sealants for Caries Prevention,” Junger et al used data from a national consumer survey to describe the lack of knowledge about dental sealants among adults and among a subgroup of adult parents of children aged 18 years or younger (23). Only about half of the respondents in each group could correctly answer a question about the purpose of dental sealants. Parents (55%) were slightly more likely than adults overall (46%) to have answered the question correctly. Disparities in sealant knowledge mirrored disparities in the use of sealants and the prevalence of caries among children: parents of children in the most disproportionately affected groups — families with less education, families with low income, and members of racial/ethnic minority populations — were less likely to be aware of the benefits of dental sealants. The authors recommend collaborative health promotion and educational efforts that draw on various groups of people, including oral health professionals, pediatricians, school nurses, and teachers. School sealant programs help reach children at high risk of caries, and these programs should be expanded.

A common theme of many of the articles in this PCD collection is the effect of access to oral health services, especially preventive services, on the prevalence of oral diseases. Access to the oral health system is the foundation for all Healthy People 2030 oral health objectives. Many oral health disparities among the populations of interest in this collection could be exacerbated by the COVID-19 pandemic. In their commentary, “Oral Health and COVID-19: Increasing the Need for Prevention and Access,” Brian and Weintraub describe disparities and opportunities in the dental community that have arisen as a result of the pandemic (24). Many oral health objectives are relevant to their discussion. Early in the pandemic, closure of dental practices except for emergencies excluded routine care and prevention. Brian and Weintraub discuss the importance of oral health during COVID-19; chronic disease disparities; access to care limitations; increased risk of infection among dental providers through use of aerosol-generating devices (ie, dental handpieces, ultrasonic scalers); opportunities for change, including the use of nonaerosol-producing dental devices, materials and procedures; increasing nonsurgical prevention and management; enhancing Medicaid reimbursement, especially the expansion of adult Medicaid dental benefits in many states where it is limited or nonexistent; easing dental workforce restrictions; and advancing teledentistry to address gaps and increase access to preventive care. One of the many negative effects of COVID-19 is that National Health and Nutrition Examination Survey activities were paused out of an abundance of caution to protect participants, survey staff members, and communities (25). The need for ongoing national surveillance of oral health and disease trends is critical because the pandemic affects access to care and care-seeking behaviors, especially in populations with pre-existing health disparities.

Although this collection of articles in PCD does not address all 11 Healthy People 2030 oral health objectives, it does touch on cross-cutting issues such as access to dental care, oral health disparities and inequities, and prevention of dental disease. Oral health objectives not addressed in this collection are to reduce the proportion of adults with active or untreated decay (objective 3), to reduce the proportion of older adults with untreated root surface decay (objective 4), to reduce the proportion of adults aged 45 or older who have lost all their teeth (objective 5), to increase the proportion of oral and pharyngeal cancers detected at the earliest stage (objective 7), to increase the proportion of people whose water systems have the recommended amount of fluoride (objective 11), and to increase number of states, plus the District of Columbia, with oral and craniofacial health surveillance systems (objective D1). Objectives developed by other Healthy People 2030 work groups also address oral health–related areas, such increasing the proportion of people with dental insurance (Access to Health Services [AHS] objective AHS-02]), reducing the proportion of people who are unable to obtain or are delayed in obtaining necessary dental care (objective AHS-05), and reducing the consumption of calories from added sugars by persons aged 2 years or older (Nutrition and Weight Status objective 10).

The second Surgeon General’s Report on Oral Health is expected to be released in 2021. The new report will describe key issues that currently affect oral health, identify challenges and opportunities that have emerged since publication of the first report, articulate a vision for the future, and call upon all Americans to take action. Its predecessor was published in 2000, with the message that oral health means much more than healthy teeth and is integral to the overall health and well-being of the US population (1). Indeed, this message continues to resonate today, because a person cannot have good overall health without having good oral health. This PCD collection of oral health articles and its underlying foundation of the Healthy People 2030 oral health objectives provides us with a roadmap for improving oral health and, thus, overall health in the United States. The articles provide a snapshot of why oral health needs to be elevated as a policy priority by being included and integrated into discussions and policy decisions about health. Thus, addressing the social, behavioral, and environmental determinants of health as part of oral health care offers a new approach to prevention and treatment (26).

## References

[1] US Department of Health and Human Services. Oral health in America: a report of the Surgeon General. Rockville (MD): US Department of Health and Human Services, National Institute of Dental and Craniofacial Research, National Institutes of Health; 2000.

[2] Cappelli D . Introduction: the advent of precision public health and the future of dental public health. J Public Health Dentistry 2020;80(Suppl 1) S5–S6. 10.1111/jphd.12359 32020614

[3] US Department of Health and Human Services, Office of Disease Prevention and Health Promotion. Healthy people 2030. https://health.gov/healthypeople. Accessed February 23, 2021.

[4] Chapple IL , Genco R ; Working Group 2 of the Joint EFP/AAP workshop. Diabetes and periodontal diseases: consensus report of the Joint EFP/AAP workshop on periodontitis and systemic diseases. J Periodontol 2013;84(4 Suppl):S106–12. 23631572 10.1902/jop.2013.1340011

[5] Ide M , Papapanou PN . Epidemiology of association between maternal periodontal disease and adverse pregnancy outcomes — systematic review. J Periodontol 2013;84(4 Suppl):S181–94. 23631578 10.1902/jop.2013.134009

[6] Dietrich T , Sharma P , Walter C , Weston P , Beck J . The epidemiological evidence behind the association between periodontitis and incident atherosclerotic cardiovascular disease. J Periodontol 2013;84(4 Suppl):S70–84. 23631585 10.1902/jop.2013.134008

[7] Kaur S , Bright R , Proudman SM , Bartold PM . Does periodontal treatment influence clinical and biochemical measures for rheumatoid arthritis? A systematic review and meta-analysis. Semin Arthritis Rheum 2014;44(2):113–22. 10.1016/j.semarthrit.2014.04.009 24880982

[8] Kamer AR , Craig RG , Dasanayake AP , Brys M , Glodzik-Sobanska L , de Leon MJ . Inflammation and Alzheimer’s disease: possible role of periodontal diseases. Alzheimers Dement 2008;4(4):242–50. 10.1016/j.jalz.2007.08.004 18631974

[9] Scannapieco FA , Bush RB , Paju S . Associations between periodontal disease and risk for nosocomial bacterial pneumonia and chronic obstructive pulmonary disease. A systematic review. Ann Periodontol 2003;8(1):54–69. 10.1902/annals.2003.8.1.54 14971248

[10] Weintraub JA , Lopez Mitnik G , Dye BA . Oral diseases associated with nonalcoholic fatty liver disease in the United States. J Dent Res 2019;98(11):1219–26. 10.1177/0022034519866442 31369716 PMC6755718

[11] Seitz MW , Listl S , Bartols A , Schubert I , Blaschke K , Haux C , Current knowledge on correlations between highly prevalent dental conditions and chronic diseases: an umbrella review. Prev Chronic Dis 2019;16:E132. 10.5888/pcd16.180641 31560644 PMC6795069

[12] US Department of Health and Human Services, Office of Disease Prevention and Health Promotion. Healthy People 2030, leading health indicators. 2020. https://health.gov/healthypeople/objectives-and-data/leading-health-indicators. Accessed December 13, 2020.

[13] Manski RJ , Rohde F . Research findings no. 38. Dental services: use, expenses, source of payment, coverage and procedure type, 1996–2015. Rockville (MD): Agency for Healthcare Research and Quality; 2017. https://meps.ahrq.gov/mepsweb/data_files/publications/rf38/rf38.pdf. Accessed February 22, 2021.

[14] Rural Health Information Hub. What oral health disparities are present in rural America? https://www.ruralhealthinfo.org/topics/oral-health#disparities. Accessed December 12, 2020.

[15] Hamilton E , Bernal J , Lin M , Thornton-Evans G , Griffin S . Visualizing county-level data to target dental safety-net programs for children. Prev Chronic Dis 2021;18:200488. 10.5888/pcd18.200488 PMC7986970 33705305

[16] Samsel S , Tramp R , Sengul Orgut I , Freeman N , Parton J , Hudnall M , Visualizing potential effects of dentist retirements on accessibility to dental care among children in Alabama, 2019. Prev Chronic Dis 2021;18:E08. 10.5888/pcd18.200410 33544073 PMC7879962

[17] Centers for Disease Control and Prevention. Oral health surveillance report: trends in dental caries and sealants, tooth retention, and edentulism, United States, 1999–2004 to 2011–2016. Atlanta (GA): Centers for Disease Control and Prevention, US Department of Health and Human Services; 2019.

[18] Robison V , Wei L , Hsia J . Racial/ethnic disparities among US children and adolescents in use of dental care. Prev Chronic Dis 2020;17:E71. 10.5888/pcd17.190352 32730202 PMC7417021

[19] Martin M , Pugach O , Avenetti D , Lee H , Salazar S , Rosales G , Oral health behaviors in very young children in low-income urban areas in Chicago, Illinois, 2018–2019. Prev Chronic Dis 2020;17:E152. 10.5888/pcd17.200213 33274700 PMC7735487

[20] Lee HH , Faundez L , Nasseh K , LoSasso AT . Does preventive care reduce severe pediatric dental caries? Prev Chronic Dis 2020;17:E136. 10.5888/pcd17.200003 33119483 PMC7665577

[21] Wright JT , Tampi MP , Graham L , Estrich C , Crall JJ , Fontana M , Sealants for preventing and arresting pit-and-fissure occlusal caries in primary and permanent molars: a systematic review of randomized controlled trials — a report of the American Dental Association and the American Academy of Pediatric Dentistry. J Am Dent Assoc 2016;147(8):631–645.e18. 10.1016/j.adaj.2016.06.003 27470524

[22] American Dental Association. MouthHealthy. Sealants. https://www.mouthhealthy.org/en/az-topics/s/sealants. Accessed March 2, 2021.

[23] Junger ML , Griffin SO , Lesaja S , Espinoza L . Awareness among US adults of dental sealants for caries prevention. Prev Chronic Dis 2019;16:E29. 10.5888/pcd16.180398 30873938 PMC6429685

[24] Brian Z , Weintraub JA . Oral health and COVID-19: increasing the need for prevention and access. Prev Chronic Dis 2020;17:E82. 10.5888/pcd17.200266 32790606 PMC7458118

[25] Centers for Disease Control and Prevention, National Center for Health Statistics. NHANES: the nation’s mobile health survey. https://www.cdc.gov/nchs/nhanes/participant.htm. Accessed January 28, 2021.

[26] US Department of Health and Human Services, Office of Disease Prevention and Health Promotion. Healthy People 2020. Oral health: using law and policy to promote the use of oral health services in the United States. https://www.healthypeople.gov/2020/law-and-health-policy/topic/oral-health. Accessed February 4, 2021.

